# Editorial: Neuroinflammation, neurodegeneration, and auditory-vestibular disorders

**DOI:** 10.3389/fneur.2025.1757515

**Published:** 2026-01-06

**Authors:** Arianna Di Stadio, Massimo Ralli, Diego Kaski

**Affiliations:** 1 Department of Life Sciences, Health and Health Profession, Link University, Rome, Italy; 2 Unicamillus International Medical School, Rome, Italy; 3 Sense Laboratory, University College of London (UCL) Queen Square Neurology, London, United Kingdom

**Keywords:** audiovestibular, neurodegeneration, neuroinflammation, pathophysiology, treatments

This Research Topic brings together contributors that explore the emerging intersections between neuroinflammation, neurodegeneration, and disorders of the auditory–vestibular system. Several of the observations highlighted in this editorial draw directly from the work presented within the Research Topic, reflecting the breadth of new evidence that positions audio-vestibular symptoms as potentially early and clinically meaningful manifestations of neuroinflammatory disease.

Audio-vestibular symptoms can represent early signs of neuroinflammation, and they might appear either at disease onset or during relapse in conditions such as Multiple Sclerosis (MS). Despite MS being a central disorder, the anatomical connection between the brain and the ear via the cochlear aqueduct, may allow passage of pro-inflammatory molecules from the cerebrospinal fluid (CSF) into the perilymph, leading to audiovestibular symptoms (Zhao et al.). The sensory receptors of the inner ear, both cochlear and vestibular, are indeed highly vulnerable to inflammation (1); even minimal concentrations of pro-inflammatory factors may induce cellular damage and lead to the loss of auditory and/or vestibular function.

Building on the connection between neuroinflammation and early sensory involvement, research presented in this Research Topic has investigated how specific cranial nerve manifestations in MRI-confirmed cases of neuro-inflammation, may help predict disease prognosis. Symptoms arising from peripheral nerve dysfunction (e.g., vertigo, hearing loss, diplopia) have been associated with better long-term outlook than cerebral or spinal inflammatory plaques, in patients with relapsing–remitting MS (Di Stadio, Scribani-Rossi et al.). Specifically, when comparing symptoms originating from the optic nerve (ON; *n* = 55) with those arising from other cranial nerves such as oculomotor, abducens, facial, or vestibulo-cochlear, (OCN; *n* = 29), MS patients with OCN symptoms showed a more favorable disease course at a 4-year follow-up.

Auditory symptoms have been associated with neurodegeneration and cognitive decline, highlighting the relevance of the ear-brain axis in Neurological disorders, with a bi-directional interplay between hearing loss and brain degeneration (2–5). In this Research Topic, Zhang et al. propose that (i) neuroinflammation and neurodegeneration certainly appear to be related and comcomitant events in some neurodegenerative diseases, including Alzheimer's disease, Parkinson's disease, and Amyotrophic lateral sclerosis, and (ii) that tinnitus may represent an early manifestation of brain neurodegeneration resulting from neuro-inflammation (6). It has been shown that tinnitus arises from hyperactivation of the auditory cortex and it is proposed that neuro-inflammation could underpin such hyperactivation (7). However, tinnitus is a common finding across the general population and therefore such a mechanism presumably only applies to a smaller cohort of susceptible individuals, although such data is lacking. Whatever the underlying mechanism, the impact of tinnitus extends beyond abnormal sound perception, leading to significant distress, cognitive dysfunction, and autonomic arousal, which complicate treatment and contribute to functional impairment (Ruan et al.). Over time, persistent tinnitus may also further exacerbate cognitive difficulties.

Indeed, microstructural changes have been linked to cognitive decline in patients with Parkinson's Disease (PD). In this Research Topic, Niu et al. report that, even in the presence of only mild cognitive impairment, patients with Parkinson's Disease (PD) show diffuse microstructural white matter abnormalities, with minimal gray matter atrophy and a predominance of changes in the left hemisphere. Subcortical structures may also be relevant to cogntive decline. A key structure bridging sensory processing and cognition is the thalamus. Its pivotal role, both as a relay and an integrative hub, means that thalamic degeneration disrupts communication across different cortical regions involved in attention, memory, executive function, and emotional regulation. In this Research Topic, Wen et al. demonstrated that the degree of thalamic atrophy is closely related to the severity of neurodegeneration, regardless of the underlying disease. The degree of thalamic involvement could thus potentially influence both the onset and progression of cognitive decline in MS, PD, Alzheimer's disease, and other dementias.

Cognitive decline is particularly prominent in the later stages of neuroinflammatory diseases such as MS, where it frequently co-occurs with severe fatigue. Both features negatively influence disability scores such as the Expanded Disability Status Scale (EDSS). Fatigue itself, a hallmark of MS and a key symptom in chronic fatigue syndromes, requires careful characterization to distinguish among neurological causes. Here, Lu et al. reviewed the psychometric and clinical utility of existing instruments for assessing persistent fatigue, post-exertional malaise, cognitive dysfunction, sleep disturbance, pain, psychological distress, orthostatic intolerance, and multidimensional health status, noting the need for more objective evaluation tools.

Given the widespread clinical burden imposed by neuroinflammation and neurodegeneration, the identification of biomarkers has become a major priority. Biomarkers can help quantify mortality risk (Ma et al.), support early detection of neuroinflammation (Tian et al.), and help finding strategies to delay or prevent neurodegenerative change (Lado et al.; Huang et al.; Wang and Zhou; Londoño et al.; Xie et al.). Neuroinflammation, driven by mechanisms such as microglial pyroptosis and inflammasome activation (Wang and Zhou), is well-described in MS and PD but may also occur transiently after vascular events such as stroke (Kim et al.). Indeed, vascular alterations themselves can trigger neuroinflammation and precipitate symptom onset (Di Stadio, Messineo et al.; Meliante et al.). Failure to adequately control brain inflammation can lead to persistent, refractory symptoms.

This expanding mechanistic understanding has catalyzed the development of novel therapeutic approaches. Several interventions show promise in modulating microglial activation and reducing neuroinflammation, including Transcranial Magnetic Stimulation (TMS) (Chan and Ng), anti-inflammatory agents (Di Stadio, Messineo et al.; Wang and Zhou), stem-cell–based therapies (Kim et al.), platelet-rich plasma (Lan et al.), and lifestyle interventions such as regular physical activity (Zhang et al.).

Together, these findings highlight the deep interconnectedness of central and peripheral sensory pathways with neuroinflammatory and neurodegenerative mechanisms. This Research Topic aims to elucidate these relationships by integrating clinical, mechanistic, and therapeutic perspectives, with the overarching goal of improving early detection, prevention, and management strategies for patients affected by auditory–vestibular and cognitive complications of neurological diseases.
